# Temporal recalibration in response to delayed visual feedback of active versus passive actions: an fMRI study

**DOI:** 10.1038/s41598-024-54660-2

**Published:** 2024-02-26

**Authors:** Konstantin Kufer, Christina V. Schmitter, Tilo Kircher, Benjamin Straube

**Affiliations:** 1 Department of Psychiatry and Psychotherapy, Philipps-University Marburg, Rudolf-Bultmann-Strasse 8, 35039 Marburg, Germany; 2 Center for Mind, Brain and Behavior (CMBB), University of Marburg and Justus Liebig University Giessen, Hans-Meerwein-Strasse 6, 35032 Marburg, Germany

**Keywords:** Auditory system, Sensorimotor processing, Sensory processing, Visual system

## Abstract

The brain can adapt its expectations about the relative timing of actions and their sensory outcomes in a process known as temporal recalibration. This might occur as the recalibration of timing between the sensory (e.g. visual) outcome and (1) the motor act (sensorimotor) or (2) tactile/proprioceptive information (inter-sensory). This fMRI recalibration study investigated sensorimotor contributions to temporal recalibration by comparing active and passive conditions. Subjects were repeatedly exposed to delayed (150 ms) or undelayed visual stimuli, triggered by active or passive button presses. Recalibration effects were tested in delay detection tasks, including visual and auditory outcomes. We showed that both modalities were affected by visual recalibration. However, an active advantage was observed only in visual conditions. Recalibration was generally associated with the left cerebellum (lobules IV, V and vermis) while action related activation (active > passive) occurred in the right middle/superior frontal gyri during adaptation and test phases. Recalibration transfer from vision to audition was related to action specific activations in the cingulate cortex, the angular gyrus and left inferior frontal gyrus. Our data provide new insights in sensorimotor contributions to temporal recalibration via the middle/superior frontal gyri and inter-sensory contributions mediated by the cerebellum.

## Introduction

In interacting with the external world, the brain is thought to predict sensory outcomes of actions by internal forward models, as a combination of the motor command’s efference copy and the current sensory state^1–5^, shaping the perception of sensory information. For the predictions to be a useful representation of the external world however, the prediction making process needs to be resilient to changes in the temporal structure of stimulation, such as the influence of lighting conditions on visual sensory processing speeds^6^. In this line, the human brain has been shown to adapt to temporal changes in action-outcome relationships in a mechanism known as sensorimotor temporal recalibration, which is achievable by inducing an artificial delay between voluntary actions and their sensory outcomes^3,4,7–9^. The consequence of temporal recalibration is known as the temporal recalibration effect (TRE) with its expression depending on the specific experimental paradigm. TREs have been shown as a worsening in performance in delay detection tasks^7,10,11^ and a modification of subjective temporal order perception of voluntary actions and sensory outcomes^9^. Temporal recalibration effects are believed to occur as an update in the prediction making process for future voluntary actions based on experienced prediction errors, indicating a mismatch between predicted and actual sensory outcomes. However, temporal recalibration has also been shown in the inter-sensory domain, as a shift of synchrony perception between sensory modalities^12,13^. Inter-sensory TREs occur in the absence of motor commands and can thus not be explained by internal forward models, suggesting recalibration mechanisms independent of sensorimotor predictions. Moreover, it seems reasonable to propose that, besides sensorimotor predictions, inter-sensory recalibration effects might play a role in sensorimotor temporal recalibration due to proprioceptive and tactile sensory information originating from the action^7,10,11^. The combination of a sensorimotor and an inter-sensory component should thereby lead to a more pronounced TRE than the inter-sensory component alone. This was already demonstrated as a greater TRE due to active opposed to externally generated actions^7,9,10^. The differential contributions of sensorimotor and inter-sensory components, however, are still largely unknown.

Natural sensory stimulation is often a complex interaction of several modalities, raising the question whether temporal recalibration might be processed on a supra-modal level. Studies suggest forward models to generate multisensory outcome predictions^14–16^, potentially allowing for a transfer of recalibration effects between senses. This notion is supported by research, which found TREs to transfer between the visual and auditory modality in both directions^17^. Conversely however, in a recent study we found no transfer of TREs from audition to vision^10^. Thus, the question of a supra-modal nature of sensorimotor temporal recalibration is not fully resolved yet.

As for the incorporation of internal forward models and temporal recalibration processes in the brain, several potentially related neural correlates were identified. Firstly, the perceived sensory intensity and neural processing of sensory outcomes of voluntary actions is reduced compared to the outcomes of externally generated actions, an effect known as sensory attenuation, which has been shown by several fMRI studies^1,18,19^. The same is true for predictably undelayed compared to unpredictably delayed outcomes of voluntary actions^3,4,14^. Interestingly, attenuation of cortex activity can be achieved for delayed sensory outcomes. This was shown in a temporal recalibration EEG study, involving auditory outcomes triggered by button presses, which found the N1 component due to delayed auditory stimulation to be reduced after delay adaptation, a pattern usually observed for undelayed auditory stimuli^4^. Recalibration of attenuation, however, has been shown to be inhibitable by disturbance of cerebellar activity through transcranial magnetic stimulation^3^. In this line, the cerebellum is proposed to encode important mechanisms of internal forward models^1,20–22^ and a recent study observed transcranial direct current stimulation (tDCS) over cerebellar regions to influence temporal recalibration^11^. This influential effect, however, occurred in sensorimotor and purely inter-sensory recalibration, indicating a role of the cerebellum in both contexts^11^. This is in line with our recent fMRI findings in delay detection tasks after auditory temporal recalibration^10^. Differential cerebellum activation, however, occurred for active compared to passive actions in the recalibration phases of the experiment^10^. Furthermore, readiness potentials in the motor system and visual cortex were shown to shift in time due to visual temporal recalibration^8^. A neural function, potentially closely related to temporal recalibration is the processing of sensory errors. This includes comparator functions, comparing sensory predictions and actual sensory outcomes, and eliciting a prediction error on a mismatch. Sensory error processing has been shown to be associated with activation in parietal areas, including the angular gyrus^16,23,24^, temporal areas^24^ and the cerebellum^25,26^.

In summary, the literature provides a variety of data on temporal recalibration and related processes. However, temporal recalibration studies including a differentiation between sensorimotor and inter-sensory components, while investigating neural correlates of recalibration are sparse, especially in the visual domain. It is still not fully resolved to what extent recalibration can be attributed to sensorimotor or inter-sensory effects. With this study, we aimed at disentangling their contribution to temporal recalibration processes by including voluntary and externally generated actions. While inter-sensory recalibration might occur with both action types, sensorimotor recalibration can only occur with voluntary actions. We used functional magnetic resonance imaging (fMRI) to acquire brain activation data. The experimental procedure was built after the action-outcome principle, while the action was comprised of a button press, either performed actively or induced passively. Each button press was followed by a sensory outcome. In adaptation phases, participants were accustomed to a fixed adaptation delay between the action and a visual sensory outcome through a repeated presentation of action-outcome pairs. In subsequent testing phases we tested the participants’ delay detection performance to assess the impact of adaptation on temporal perception. To investigate within-modality and cross-modality TREs, we included visual and auditory sensory outcomes in testing phases. We recently used a comparable experimental procedure in a temporal recalibration study in the auditory sensory domain^10^.

We expected the adaptation delay to have a significant impact on temporal perception, resembled by a worsening in delay detection performance after temporal recalibration. Based on the hypothesis that temporal recalibration using voluntary actions involves a sensorimotor component in addition to a more general inter-sensory component we expected a greater TRE for active compared to passive button presses in within-modal conditions^7,10^. As for neural correlates, we expected regions thought to be associated with sensorimotor temporal recalibration and internal forward models, including the cerebellum, to be associated with recalibration, especially in active conditions. We expected regions, known to be involved in more general inter-sensory error processing, including temporal and parietal areas, to activate for recalibration in active and passive conditions. However, the complexity of our experimental approach with highly controlled varying conditions most likely requires the recruitment of several brain regions, rather than being mediated by a few. Thus, due to the complex nature of the experimental task and the novelty of our approach, we performed a whole-brain, rather than a restrictive, fMRI-data analysis. Finally, based on the notion of a supra-modal processing of sensorimotor predictions, we expected a significant TRE in auditory testing phases, especially in active conditions.

## Materials and methods

### Participants

Twenty-four healthy subjects volunteered to participate in the study (10 females, 14 males; Mean age: 24.8 years, SD = 5.24 years). All had normal or corrected to normal eyesight and reported righthandedness (additionally quantified by the Edinburgh handedness questionnaire^27^, 10-item: mean laterality quotient = 80.0%, SD = 23.3). No acute or past psychiatric or neurological disorders were reported. All subjects received financial remuneration and gave written informed consent to their participation. The study was conducted in accordance with the declaration of Helsinki (except for pre-registration) and was approved by the local ethics commission of the Faculty of Medicine at the Philipps-University of Marburg.

### Equipment

**Figure 1:**
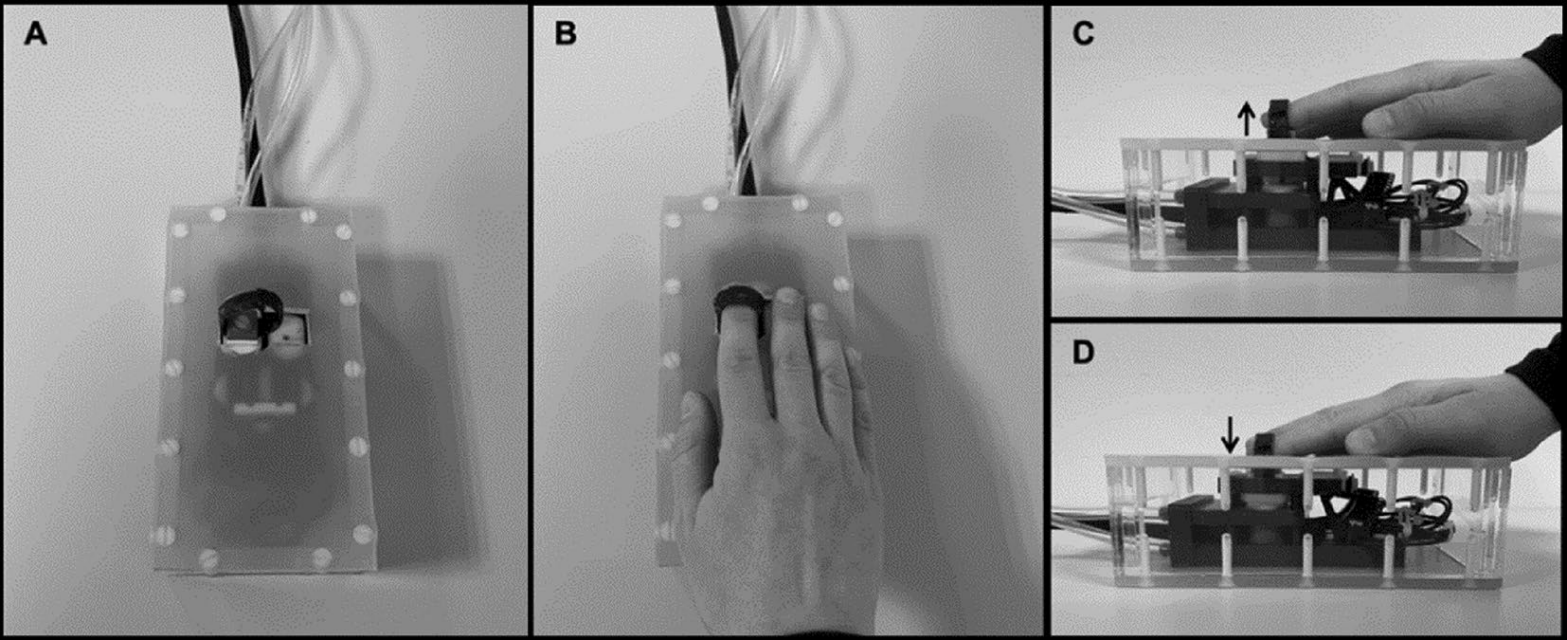
Custom-made pneumatic MR-compatible button; as in reference^10^ (**A**) Top view on the button. (**B**) Hand and finger-positioning on the button. (**C**) Relaxed, non-pressed button position. (**D**) Fully pressed button.

Core equipment was a custom-made pneumatic MR-compatible button, placed on the participant’s right side, with the right index finger on top of the button (see Fig. 1). The button was connected to a pneumatic system, enabling experimentally controlled button presses by forcing the button to move down, mimicking an active button press (1 bar pressure; 20N max. force). In addition, the participant’s finger was strapped to the button with a fabric band, to ensure movement synchronization of button and finger. This allowed for active button presses by the participants and passive button presses by the pneumatic system with similar tactile and proprioceptive stimulation. Button movements were tracked through a light barrier system using optic fibres. A monitor screen (60 Hz refresh rate) was placed behind the MRI scanner, visible through mirrors attached to the MRI-head coil equipment. Visual stimulation (Gabor patch: 1-degree visual angle; 2 cycles/degree spatial frequency) and written instructions, were presented on the monitor. Auditory stimuli (sine-wave tone: 2000 Hz; 2 ms rise and fall) were presented over MR-compatible Headphones (MR-Confon Optime1, Magdeburg, Germany). Each button press was followed by a sensory outcome represented by a visual or auditory stimulus. This sensory stimulation was triggered by the button reaching the fully pressed state and the stimulus was presented for 33.3 ms in all cases. The stimulus onset could be delayed by a duration of up to 417 ms, as an additional delay to the inherent technical processing time in the equipment. The participants were able to indicate whether they detected a delay during the delay detection task through a “response keyboard”, located on their left side.

### Experimental design

**Figure 2:**
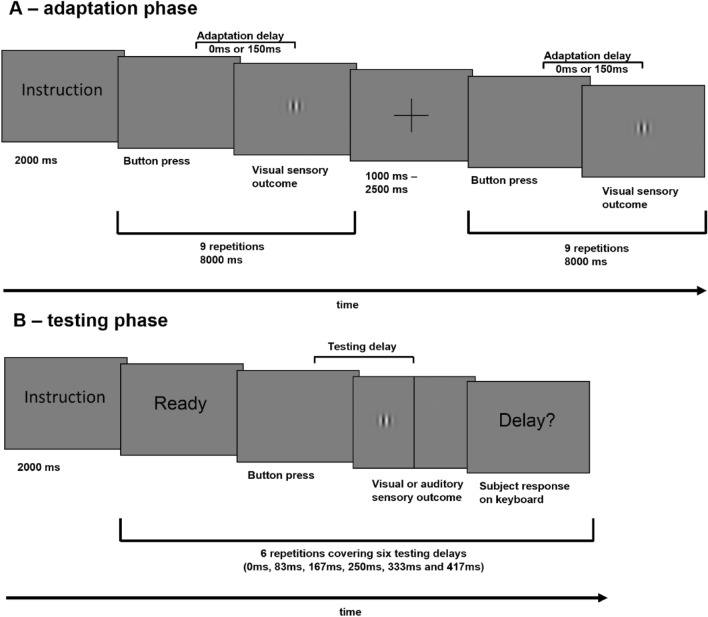
Experimental task. (**A**) Adaptation phase. Starting with instructions (2000 ms), depicting *movement agency*. First button pressing part after disappearing of instructions (max. 8000 ms). Button presses with visual sensory outcome, either delayed or undelayed (*adaptation delay*). Short pause after, with jittered duration (1000 ms, 1500 ms, 2000 ms, 25,000 ms) while presenting a fixation cross. Second button pressing part after disappearance of cross (max. 8000 ms). (**B**) Testing phase. Starting with instructions (2000 ms), depicting *movement agency* and *sensory testing modality*. Single button press after a “ready” cue. Sensory outcome was delayed by one of six testing delays (0 ms, 83 ms, 167 ms, 250 ms, 333 ms, 417 ms). “Delay?” question appeared after button press and participants answered with yes, delay detected or no, no delay detected. Six repetitions per block, covering all six testing delays.

The task was blocked into multiple sequences of adaptation (see Fig. 2A) and testing phases (see Fig. 2B). During each block the button was repeatedly pressed, while every press was followed by a single sensory outcome. The factor *movement agency* defined whether the button was pressed actively or passively. The *movement agency* did not vary throughout a single block and was thus the same in corresponding adaptation and testing phases. In adaptation phases, the button press action was repeatedly performed with visual sensory outcomes. The sensory outcome was either delayed or undelayed, defined as the *adaptation delay* (150 ms/0 ms). This phase was not communicated as an “adaptation” phase, to ensure naivety in terms of outcome delays. The participants’ delay detection performance was assessed in testing phases, separated into six consecutive trials. The button was pressed once per trial, with a delayed outcome. Six testing delays were defined (0 ms, 83 ms, 167 ms, 250 ms, 333 ms, 417 ms). Each testing delay was presented once per testing phase, with the order of presentation counterbalanced across all blocks. After a testing trial, the participants reported whether they detected a delay or not by pressing a predefined key on the response keyboard. The key assignment (yes/no) was counterbalanced across participants. In contrast to adaptation phases, the sensory outcome in testing phases was either visual or auditory, defining the *sensory testing modality*. The combination of the three binary factors *adaptation delay* (0 ms/150 ms), *movement agency* (active/passive) and *sensory testing modality* (visual/auditory) resulted in eight different conditions. The procedure was separated into four runs, sixteen blocks each. Consequently, every condition was covered twice per run and eight times in total per participant. The *adaptation delay* was switched once per run, resulting in eight consecutive blocks per delay. Within these eight consecutive blocks, the *movement agency* was switched once, resulting in two groups of four blocks. The order of *adaptation delay* and *movement agency* conditions was counterbalanced across runs and participants. The participants were familiarised with the procedure in a training session in advance to the fMRI session.

Each block started with an adaptation phase and written instructions presented for 2000 ms, depicting the current phase and *movement agency* condition. As the instructions disappeared, the button was repeatedly pressed. Each press triggered a visual sensory outcome either delayed or undelayed. The button pressing was separated into two parts, each lasting 8000 ms at maximum, divided by a fixation cross with jittered duration (1000 ms, 1500 ms, 2000 ms, 2500 ms), representing a short pause. In passive conditions the button presses were performed with a 500 ms duration and an 800 ms interval, with nine presses per part within the 8000 ms time frame. For active conditions, the participants were trained to the same durations and intervals in the training session, resulting in nine active button presses per part, as in passive conditions, when correctly executed. Active adaptation phase parts ended after 8000 ms or after the ninth button press was fully executed. After the second part, the testing phase began, starting with written instructions, reminding of the current *movement agency* and depicting the following *sensory testing modality*. As the instructions disappeared, six consecutive testing trials began. Each trial started with a written “ready” cue presented for 1000 ms. As the cue disappeared the button was pressed once within a 2000 ms time frame. The participants were instructed to pause for approximately 700 ms before pressing the button in active conditions, to ensure a consciously initiated action, rather than a reflex tied to the disappearance of the “ready” cue. In passive conditions, this initiation pause was experimentally controlled with jittered duration (0 ms, 500 ms, 1000 ms). The button press triggered a sensory outcome, delayed by one of the testing delays. After the 2000 ms button press time frame elapsed, the written question “delay?” appeared and the participants gave their response on the response keyboard (delay detected: yes/no) within 2000 ms. This button press time frame in combination with the button press initiation pause led to a jittered relative timing between time frame onset and the button press, as well as the button press and the time frame end. After a 500 ms pause, the next “ready” cue appeared, repeating the testing procedure for all six trials. The following block’s onset was paused for a jittered duration (1000 ms, 1500 ms, 2000 ms, 2500 ms).

### fMRI data acquisition

The magnetic resonance imaging data were obtained using a Siemens (Erlangen Germany) 3 Tesla MR Magnetom TIM Trio scanner at the Department of Psychiatry and Psychotherapy in Marburg, utilizing a 12-channel head coil. Functional images were acquired with a T2*-weighted gradient echo-planar imaging sequence (64 × 64 matrix; repetition time (TR) = 1650 ms; time to echo (TE) = 25 ms; flip angle = 70°; slice thickness = 4 mm; gap size = 15%; voxel size = 3 × 3 × 4.6 mm; field of view (FOV) = 192 mm). Functional image slices were obtained parallel to the intercommissural plane, covering the whole brain, in descending order. This resulted in a total of 560 volumes per run and a slice count of 34 per volume. Anatomical images were acquired using a T1-weighted MPRAGE sequence (256 × 256 matrix; repetition time (TR) = 1900 ms; time to echo (TE) = 2.26 ms; flip angle = 9°; slice thickness = 1 mm; gap size = 50%; field of view (FOV) = 256 mm; voxel size = 1 × 1 × 1.5 mm), covering the whole brain. The whole cerebellum was covered by the recording volume.

### Data analysis

Data originating from incorrectly executed button pressing were modelled as regressors of no interest in the fMRI analysis and excluded from the behavioural analysis. This included single test trials with incorrect button presses (1.5% of test trials) and whole blocks, with less than three correct button presses in either the first or second part of adaptation, while nine correct button presses were expected per part in the adaptation phases (0.52% of blocks). Test trials with no response (7.5% of test trials) were excluded from the behavioural analysis.

#### Behavioural data analysis

For each subject and condition, the proportion of detected delays in relation to the delay level was determined. Based on this data, psychometric functions of behaviour were calculated based on a cumulative gaussian distribution function, using the psignifit toolbox^28^ in a python 3.7 environment. Under the assumption of a similar probability of rejecting the correct answer at both extreme ends of testing delays, the psychometric functions asymptotes were yoked. The lower asymptote represented the “guess” rate, guessing a delay was present. The “lapse” rate defines the rejection of the highest, most noticeable delay and represents the upper asymptote. Based on the psychometric function, the delay detection threshold was determined, defined as the delay level with 50% of delays detected. The threshold served as a measure of delay detection performance, while low thresholds indicate good performances. The slopes of the psychometric functions were determined at the delay detection thresholds. The TRE was defined as the impact of the *adaptation delay* and was calculated as the difference in delay detection thresholds between 150 and 0 ms conditions. A positive TRE resembles a shift into higher delay levels, indicating a worsening in performance. Three-way repeated measures ANOVAs with the factors *adaptation delay*, *movement agency* and *sensory testing modality* were performed on the delay detection thresholds and slope values, respectively. All significant interaction effects in the ANOVAs were post-hoc tested through pairwise t-tests. Additionally, each condition was tested for a significantly positive TRE through a one-sample one-sided t-test. Differences in TREs between conditions were tested for significance through paired t-tests. The ANOVAs were calculated using the statistical analysis program R, while t-tests were performed in python, using the SciPy software library in a python 3.7 environment.

#### fMRI data analysis

The fMRI data were pre-processed and analysed using the Statistical Parametric Mapping software (SPM12; https://www.fil.ion.ucl.ac.uk/spm/) in a Matlab environment version R2017a (MathWorks; Massachusetts). Functional images were realigned to the mean image per run, to account for head movement during data acquisition. Anatomical images were co-registered to the functional images, segmented and normalized to the Montreal Neurological Institute (MNI) template. Functional images were normalized to the MNI template, resampled to a 2 × 2 × 2 mm voxel size and smoothed with an 8 mm full width at half maximum (FWHM) kernel.

On the first level, a multiple regression analysis was performed for each subject individually. For adaptation phases, eight regressors of interest were defined, representing the conditions comprised of the binary factors *adaptation delay* and *movement agency*, modelled for the first and second part of adaptation separately. The regressors were defined from beginning to end of the adaptation parts, each (approx. 8 s duration; Active: Mean = 7.48 s, SD = 0.722 s; Passive: Mean = 7.97 s, SD = 0.185 s). For testing phases, eight regressors of interest were defined, representing the conditions comprised of the binary factors *adaptation delay*, *movement agency* and *sensory testing modality*. The regressors were defined for each testing trial, in which the button was pressed once, from beginning to end, each (approx. 2 s duration). Presentations of texts (instructions, ready cue), the “response” phase in which the question cue was displayed and left-hand responses were given, and the fixation cross were included as regressors of no interest, defined from presentation beginning to end. Two additional regressors of no interest were defined, one for adaptation phases and one for testing phases, in which the button pressing was incorrectly executed. To account for head movement, the six movement parameters, acquired in the realignment pre-processing, were included as nuisance regressors. A high-pass filter (128 s cut-off period) was applied to filter fluctuations with a low frequency (< 0.0078 Hz) to correct for technical baseline drift. All regressors of interest and of no interest were modelled using the canonical hemodynamic response function. Each regressor of interest was contrasted against the implicit baseline, resulting in sixteen first-level baseline contrasts per subject.

On the group level, a flexible factorial design was established with an independent “subject” factor (equal variance) and a dependent “condition” factor (unequal variance). This resulted in a matrix of 16 conditions × 24 participants, filled with the baseline-contrasts obtained in the first-level analysis. Significant differences in brain activation were calculated through t-tests, contrasting conditions against each other. Since fMRI contrast creation involves multiple comparisons that have to be accounted for, a Monte Carlo simulation^29,30^ was performed to find the cluster extent at which the probability for a false-positive cluster is below an alpha threshold of 0.05 and can thus be classified as significant activation. The simulation was performed with 10,000 iterations and an estimated data smoothness of 9 mm, suggesting that a cluster extent of 63 voxels is sufficient to correct for multiple comparisons, when applying a cluster forming threshold of *p* < 0.001. The resulting group-level data were labelled using the Automated Anatomic Labelling toolbox version 3^31–33^ for the Statistical Parametric Mapping software (SPM12).

The main effect of the *adaptation delay* factor and the interaction between the *adaptation delay* and *movement agency* were computed in both directions for adaptation and testing phases separately. Commonalities in activations between phases were assessed through conjunction analyses. The interaction between the *adaptation delay* and the *sensory testing modality* and the triple interaction between the *adaptation delay*, the *movement agency*, and the *sensory testing modality* were calculated for testing phases.

### Ethical approval

The study was approved by the local ethics commission (Study 141/17) of the medical faculty of University of Marburg, Germany.

## Results

### Behavioural results

**Figure 3:**
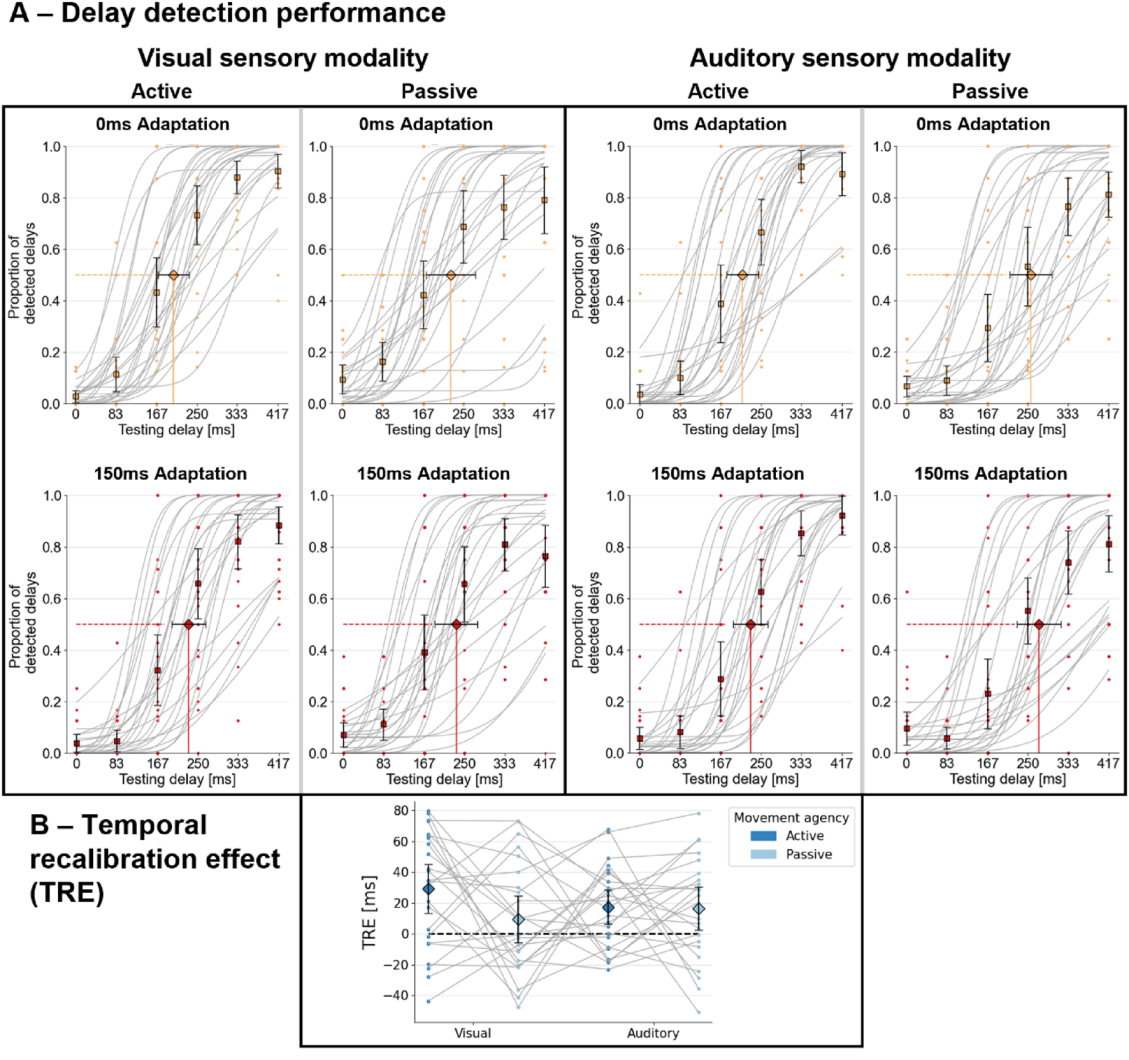
Behavioural results. (**A**) Delay detection performances in each of the eight conditions. Individual psychometric functions per participant are shown in grey. Dots indicate individual data points per participant. Squared markers indicate average datapoints per testing delay level. Diamond markers indicate average detection thresholds (50%) extracted from the individual psychometric functions. (**B**) Temporal recalibration effects (TREs) defined as 150 ms adaptation thresholds minus 0 ms adaptation thresholds. Data points indicate individual participant data. Diamond markers indicate average TREs. Grey lines connect data points of the same participant. (A/B) Error-bars represent 95% confidence intervals.

According to the repeated-measures ANOVA the *adaptation delay* significantly impacted the delay detection thresholds (*F*(1,23) = 26.4; *p* < 0.001, *η*_*p*_^2^ = 0.535) with an increase due to delayed adaptation compared to undelayed adaptation quantified by the TRE (Mean = 18.1 ms, SD = 33.6 ms). There was no significant interaction between the *adaptation delay* and the *sensory testing modality* (*F*(1,23) = 0.142; *p* = 0.710; *η*_*p*_^*2*^ = 0.006), indicating similar TREs in within-modality, visual and cross-modality, auditory conditions. In addition, there was no significant interaction between the *adaptation delay* and the *movement agency* (*F*(1,23) = 2.09; *p* = 0.162, *η*_*p*_^*2*^ = 0.083), indicating similar overall TREs in active and passive conditions. As a further in-depth analysis of temporal recalibration in different conditions, pairwise t-tests were performed on the TREs within conditions. Regarding visual testing phases, the TRE was significantly positive in active (Mean = 29.1 ms, SD = 36.6 ms; *t*(23) = 3.82, *p* < 0.001, *d* = 0.780, one-tailed), but not passive conditions (Mean = 9.49 ms, SD = 35.2 ms; *t*(23) = 1.29, *p* = 0.105, *d* = 0.263, one-tailed). The active advantage (active > passive) in TREs reached significance (*t*(23) = 1.86, *p* = 0.038, *d* = 0.379, one-tailed), supporting the assumption of the sensorimotor component’s relevance in temporal recalibration, at least for within-modality recalibration. Regarding auditory, cross-modality trials, a significantly positive TRE was observed in both, active (Mean = 17.3 ms, SD = 25.9 ms; *t*(23) = 3.21, *p* = 0.002, *d* = 0.654, one-tailed) and passive (Mean = 16.3 ms, SD = 32.5 ms; *t*(23) = 2.40; *p* = 0.012, *d* = 0.490, one-tailed) conditions and their difference was non-significant (*t*(23) = 0.121, *p* = 0.905, *d* = 0.025). Thus, in the case of cross-modality trials, no contribution of the sensorimotor component was evident. This seemingly indicates a difference in the sensorimotor component contribution between within-modality and cross-modality conditions. However, this difference did not reach significance in the repeated measures ANOVA as the triple interaction between the *adaptation delay*, the *movement agency,* and the *sensory testing modality* (*F*(1,23) = 2.05; *p* = 0.166, *η*_*p*_^*2*^ = 0.082), suggesting the observed TREs to be mainly driven by the inter-sensory component (see Fig. 3 for illustrations).

A three-way repeated measures ANOVA was performed on the slope values of the psychometric functions. A change in slope values would indicate a modification in the discriminability of testing delays. No significant effects were found for neither the main effect of the *adaptation delay* (*F*(1,23) = 1.93, *p* = 0.178, *η*_*p*_^*2*^ = 0.077), nor the double interactions of the *adaptation delay* with the factor *movement agency* (*F*(1,23) = 0.004, *p *= 0.952, *η*_*p*_^*2*^ < 0.001) or the factor *sensory testing modality* (*F*(1,23) = 0.385, *p* = 0.541, *η*_*p*_^*2*^ = 0.016), respectively. Furthermore, the triple interaction between all factors did not reach significance (*F*(1,23) = 0.012, *p* = 0.912, *η*_*p*_^*2*^ < 0.001). Thus, the delay during adaptation phases did not modify the discriminability between delay levels in testing phases.

### fMRI results

#### Main effect: *adaptation delay*

**Figure 4:**
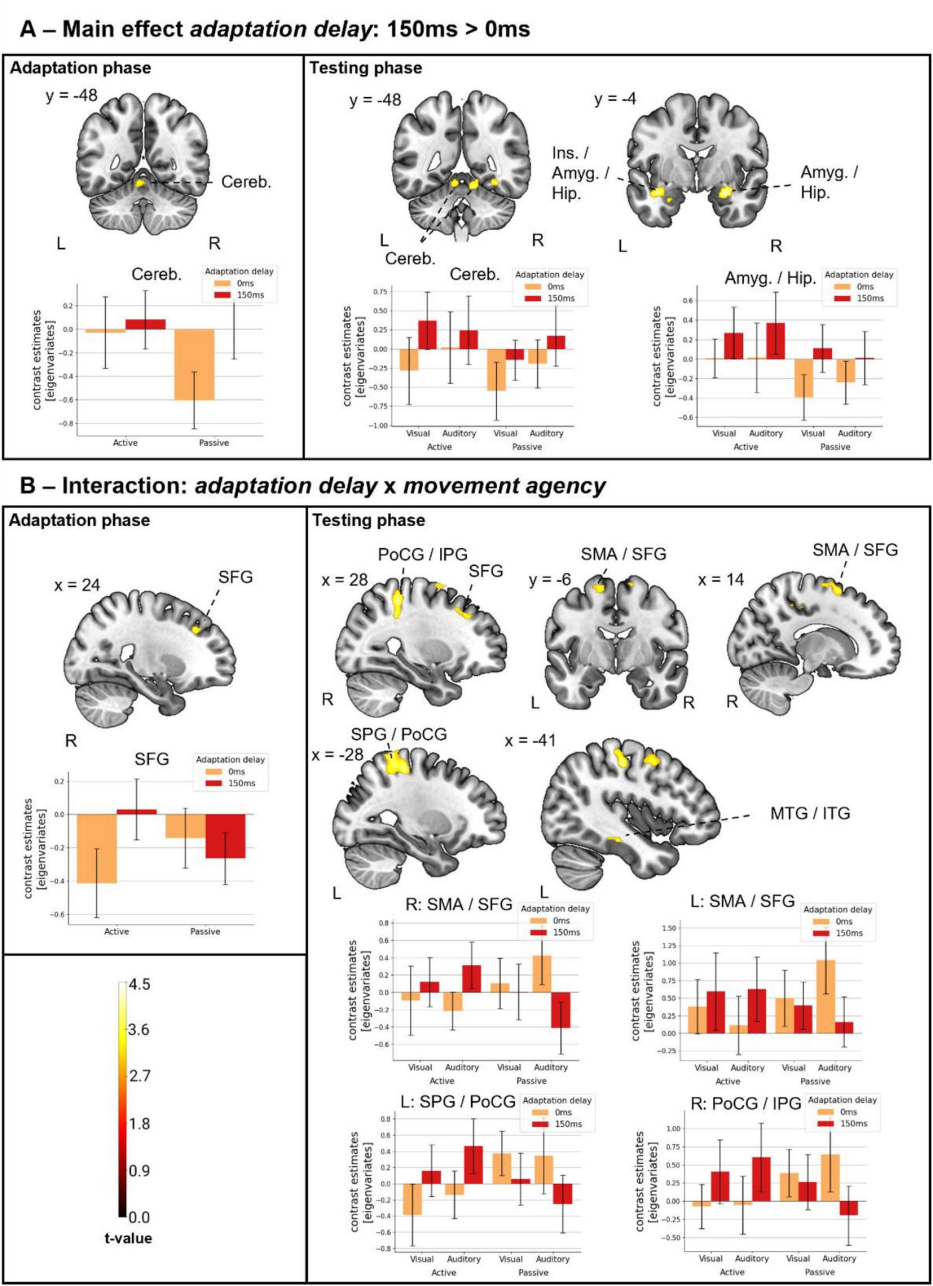
Neural correlates of temporal recalibration. (**A**) Main effect of adaptation delay. Increased activation due to temporal recalibration (150 ms > 0 ms) in adaptation phases and testing phases as brain overlays. (**B**) Interaction of adaptation delay and movement agency. Increased activation due to active temporal recalibration (Active: 150 ms > 0 ms) and decreased activation due to passive temporal recalibration (Passive: 0 ms > 150 ms) as brain overlays. (A/B) Bar plots show condition contribution to activation in a selection of clusters (largely similar pattern in all clusters), represented by contrast estimates [eigenvariates]. Error-bars represent 95% confidence intervals. Colour bar (bottom, left) depicting t-value colour coding for brain overlays. L./R.: left/right hemisphere; Cereb.: Cerebellum; Ins.: Insula; Amyg.: Amygdala; Hip.: Hippocampus; PoCG: Postcentral gyrus; IPG: Inferior parietal gyrus; SFG: Superior frontal gyrus; SMA: Supplementary motor area; SPG: Superior parietal gyrus; MTG: Middle temporal gyrus; ITG: Inferior temporal gyrus.

The main effects of the *adaptation delay* were calculated as contrasts comparing delayed adaptation conditions and undelayed adaptation conditions in both directions. This analysis revealed increased activation due to temporal recalibration (*adaptation delay*: 150 ms > 0 ms) in adaptation and testing phases (see Fig. 4A and Table 1 for detailed results). For adaptation phase this included a single activation cluster extending over the left cerebellum (Lobules IV, V) and the vermis (Lobules IV, V). For testing phases, the analysis most notably revealed activation in the cerebellum (Lobules IV, V; bilateral), the vermis (Lobules IV, V) and the hippocampus (bilateral). A conjunction analysis revealed activations to overlap between adaptation and testing phases in 40 voxels in a cluster extending over the left hemisphere of the cerebellum (lobules IV, V) and the vermis (lobules IV, V). The reverse contrasts (*adaptation delay*: 0 ms > 150 ms) revealed no significant activation for either phase.

**Table 1:** Cluster data for main effect *adaptation delay.*

Peak	Extent	Percent	Hem	Coords x; y; z	Cluster size	t-value (peak)
Adaptation phase: 150 ms > 0 ms						
Cerebellum IV, V	Cerebellum IV, V	69.23	L	− 6; − 48; − 12	65	3.6
	Vermis IV, V	30.77	–			
Testing phase: 150 ms > 0 ms						
Amygdala	Amygdala	67.24	R	28; − 4; − 18	116	4.31
	Hippocampus	19.83	R			
Superior temporal gyrus	Insula	10.74	L	− 40; − 6; − 18	149	4.17
	Amygdala	6.71	L			
	Hippocampus	4.03	L			
	Middle temporal gyrus	1.34	L			
	Superior temporal gyrus	1.34	L			
Fusiform	Fusiform gyrus	55.7	R	32; − 58; − 2	158	4.03
	Lingual gyrus	2.53	R			
Cerebellum IV, V (R)	Cerebellum IV, V	51.64	R	12; − 48; − 16	122	3.88
	Cerebellum IV, V	28.69	L			
	Vermis IV, V	18.03	–			
	Lingual gyrus	1.64	R			
Conjunction: 150 ms > 0 ms						
Cerebellum IV, V	Cerebellum IV, V	75.00	L	− 6; − 48; − 12	40*	3.6
	Vermis IV, V	25.00	–			

Hem.: Hemisphere; L./R.: left/right hemisphere; Coords.: MNI-Coordinates (peak); Percent.: percentage of voxels in label; “outside” percentages were excluded. *The cluster of the conjunction analyses is smaller than the cluster threshold (63 voxels) but indicates the overlap of the two independently significant contrasts in at least 40 voxels.

The main effects of the *adaptation delay* did not take the differential conditions into account. Thus, the found activations were not specific to either a combined sensorimotor and inter-sensory context (active button presses) or a purely inter-sensory context (passive button presses). Moreover, they were not specific to either within-modality, visual or cross-modality, auditory testing phases.

#### Interaction: *adaptation delay* and *sensory feedback modality*

To analyse for differential processing of the *sensory feedback modality* after temporal recalibration, the interaction between the *adaptation delay* and the *sensory feedback modality* during testing phases was calculated. Since this analysis was performed across both *movement agencies* the results represent a more general inter-sensory processing of temporal recalibration. However, no significant activation was found in either direction or phase.

#### Interaction: *adaptation delay* and *movement agency*

Differential processing of temporal recalibration depending on the *movement agency* was analysed as the interaction between the *adaptation delay* and the *movement agency* factors. For both, adaptation phases and testing phases, increased activation due to active temporal recalibration (*adaptation delay*: 150 ms > 0 ms) but decreased activation due to passive temporal recalibration (*adaptation delay*: 0 ms > 150 ms) was found (see Fig. 4B and Table 2). For adaptation phases, this activation included a single activation cluster in the right frontal lobe (right superior/middle frontal gyri). For testing phases, activation was most notably found in the parietal lobe (bilateral postcentral gyri, bilateral superior/inferior parietal gyri), temporal lobe (left middle/inferior temporal gyri), frontal lobe (right superior/middle frontal gyri, left superior frontal gyrus, bilateral supplementary motor areas, bilateral precentral gyri) and paracentral lobule (bilateral). Interestingly, in an exploratory analysis of the same interaction computed for visual and auditory conditions in isolation, the pattern reached significance in auditory testing phases only. This indicates that the overall activation mainly stemmed from auditory testing phases, although the pattern was present in both modalities in the overall interaction. A conjunction analysis revealed an overlap of 23 voxels between adaptation and testing phases in the superior and middle frontal gyri cluster. The reverse contrast revealed no significant activation in either phase.

**Table 2:** Cluster data for interaction effect *adaptation delay* × *movement agency.*

Peak	Extent	Percent	Hem	Coords x; y; z	Cluster size	t-value (peak)
Adaptation phase: adaptation delay × movement agency						
Superior frontal gyrus	Middle frontal gyrus	54.55	R	24; 30; 36	66	3.74
	Superior frontal gyrus	45.45	R			
Testing phase: adaptation delay × movement agency						
Superior parietal gyrus	Postcentral gyrus	68.84	L	− 28; − 46; 72	860	4.49
	Superior parietal gyrus	17.67	L			
	Precentral gyrus	9.07	L			
	Inferior parietal gyrus	2.44	L			
Paracentral lobule	Middle cingulate gyrus	45.42	L	− 10; − 26; 52	284	4.25
	Paracentral lobule	27.46	L			
	Supplementary motor area	9.15	L			
Postcentral gyrus	Postcentral gyrus	37.91	R	30; − 40; 54	728	4.17
	Inferior parietal gyrus	27.2	R			
	Supra marginal gyrus	8.79	R			
	Superior parietal gyrus	6.46	R			
	Middle cingulate gyrus	3.16	R			
	Paracentral lobule	1.37	R			
	Praecuneus	0.69	R			
	Supplementary motor area	0.41	R			
Middle frontal gyrus	Middle frontal gyrus	75.79	R	38; 20; 50	409	4.16
	Precentral gyrus	18.34	R			
	Superior frontal gyrus	3.67	R			
Precentral gyrus	Precentral gyrus	75.86	L	− 48; 6; 50	116	4.16
	Middle frontal gyrus	24.14	L			
Inferior temporal gyrus	Middle temporal gyrus	52.11	L	− 42; − 28; − 18	71	3.96
	Inferior temporal gyrus	40.85	L			
Supplementary motor area	Supplementary motor area	47.75	R	14; 8; 62	178	3.96
	Superior frontal gyrus	46.63	R			
	Middle frontal gyrus	1.12	R			
Supplementary motor area	Supplementary motor area	57.84	L	0; 22; 46	102	3.9
	Superior frontal gyrus, medial	25.49	L			
	Superior frontal gyrus, medial	8.82	R			
	Supplementary motor area	5.88	R			
	Middle cingulate gyrus	1.96	R			
Inferior frontal gyrus, opercular	Inferior frontal gyrus, opercular	63.16	R	46; 8; 22	209	3.88
	Inferior frontal gyrus, triangular	27.27	R			
	Precentral gyrus	0.96	R			
Superior frontal gyrus	Superior frontal gyrus	37.14	L	− 18; 10; 48	70	3.79
	Middle cingulate gyrus	8.57	L			
	Supplementary motor area	5.71	L			
	Middle frontal gyrus	1.43	L			
Supplementary motor area	Supplementary motor area	73.61	L	− 16; − 6; 66	72	3.57
	Superior frontal gyrus	13.89	L			
	Precentral gyrus	8.33	L			
Conjunction: adaptation delay × movement agency						
Middle frontal gyrus	Middle frontal gyrus	95.65	R	28; 30; 38	23*	3.49
	Superior frontal gyrus	4.35	R			

Hem.: Hemisphere; L./R.: left/right hemisphere Coords.: MNI-Coordinates (peak); Percent.: percentage of voxels in label; “outside” percentages were excluded. *The cluster of the conjunction analyses is smaller than the cluster threshold (63 voxels) but indicates the overlap of the two independently significant contrasts in at least 23 voxels.

#### Interaction: *adaptation delay*, *movement agency* and *sensory testing modality*

**Figure 5:**
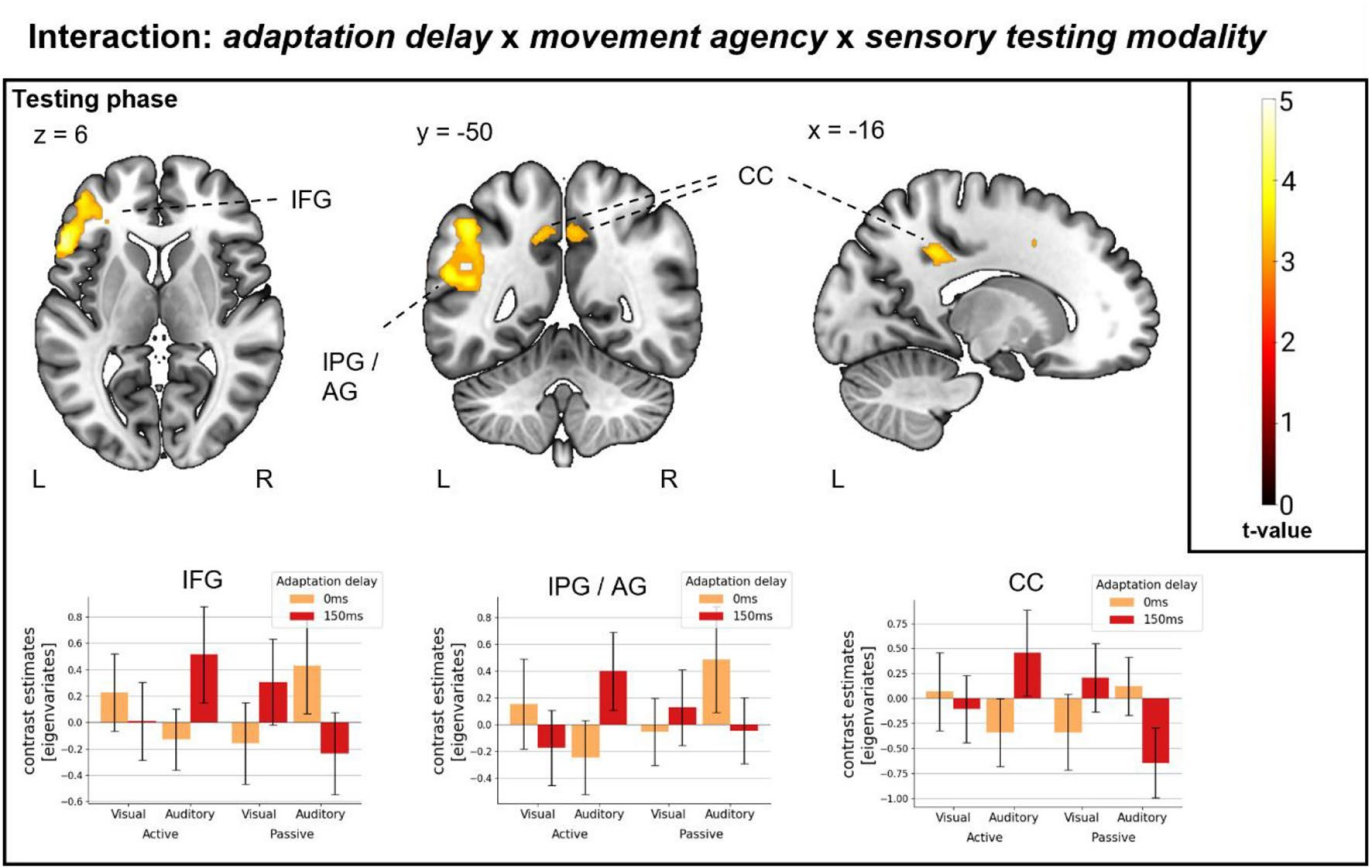
Triple interaction. Triple interaction: *adaptation delay* x *movement agency* x *sensory testing modality* (Auditory: active: 150 ms > 0 ms, passive: 0 ms > 150 ms; Visual: active: 0 ms > 150 ms, passive: 150 ms > 0 ms). Bar plots show condition contribution to activation in a selection of clusters (largely similar pattern in all clusters), represented by contrast estimates [eigenvariates]. Error-bars represent 95% confidence intervals. Colour bar (right) depicting t-value colour coding for brain overlays. L./R.: left/right hemisphere; IPG.: Inferior parietal gyrus; IFG.: Inferior frontal gyrus; AG.: Angular gyrus; CC.: Cingulate cortex.

A triple interaction between the *adaptation delay,* the *movement agency,* and the *sensory testing modality* was observed. Thereby, for auditory testing phases an increased activation due to active temporal recalibration (*adaptation delay*: 150 ms > 0 ms) but a decreased activation due to passive temporal recalibration (*adaptation delay*: 0 ms > 150 ms) was found, while the pattern was reversed for visual testing phases. This pattern included activation in the left frontal lobe (left inferior frontal gyrus), the left parietal lobe (left angular gyrus) and posterior areas in the cingulate cortex (bilateral middle/posterior cingulate gyri). No activation was found in the reverse interaction (see Fig. 5 and Table 3 for detailed results).

**Table 3:** Cluster data for interaction effect *adaptation delay* × *movement agency* × *sensory testing modality.*

Peak	Extent	Percent	Hem	Coords x; y; z	Cluster size	t-value (peak)
Interaction: adaptation delay × movement agency × sensory testing modality						
Middle cingulate gyrus	Middle cingulate gyrus	2.65	R	24; − 16; 40	151	4.65
Inferior frontal gyrus, triangular	Inferior frontal, triangular	56.54	L	− 54; 24; 6	787	4.6
	Middle frontal gyrus	18.04	L			
	Inferior frontal pars orbitalis	9.53	L			
	Superior frontal gyrus	6.86	L			
	Inferior frontal gyrus, opercular	6.73	L			
	Precentral gyrus	0.25	L			
Middle frontal gyrus	Precentral gyrus	7.76	L	− 26; 2; 42	116	4.26
	Superior frontal gyrus	6.03	L			
	Middle frontal gyrus	0.86	L			
Inferior parietal gyrus	Inferior parietal gyrus	39.51	L	− 46; − 50; 46	486	4.09
	Supra marginal gyrus	18.72	L			
	Angular gyrus	14.4	L			
	Middle temporal gyrus	13.79	L			
	Superior temporal gyrus	6.17	L			
Middle cingulate gyrus	Praecuneus	20.58	R	− 16; − 44; 38	379	3.74
	Middle cingulate gyrus	18.73	R			
	Praecuneus	14.51	L			
	Middle cingulate gyrus	13.19	L			
	Posterior cingulate gyrus	9.76	L			
	Posterior cingulate gyrus	5.8	R			
Fusiform gyrus	Inferior occipital gyrus	66.15	R	42; − 76; − 18	65	3.7
	Cerebellar hemisphere, Crus I	18.46	R			
	Fusiform gyrus	7.69	R			
	Lingual gyrus	3.08	R			

Hem.: Hemisphere; L./R.: left/right hemisphere Coords.: MNI-Coordinates (peak); Percent.: percentage of voxels in label; “outside” percentages were excluded.

## Discussion

In this study, we investigated the impact of visual temporal recalibration on temporal action-outcome perception. Our behavioural data suggest that the TRE was mainly reliant on an inter-sensory component, with an additional contribution of the sensorimotor component to the within-modal TRE. The encoding and retrieval of newly learned temporal relationships in temporal recalibration was generally related to activation in the cerebellum (lobules IV, V) and vermis (Lobules IV, V), with action dependant activation (active > passive) in the right middle/superior frontal gyri. In addition, the recall of learned relationships was associated with condition-dependant changes in task affordance during the delay detection task related to general activation in the bilateral hippocampus and action dependant activation (active > passive) in the parietal lobe (bilateral postcentral gyri, bilateral superior/inferior parietal gyri), temporal lobe (left middle/inferior temporal gyri) and frontal lobe (bilateral supplementary motor areas, bilateral precentral gyri). The transfer of TREs from vision to audition is solely explainable by the inter-sensory component on the behavioural level. However, our fMRI data point to a more complex pattern with several action specific effects, with posterior areas in the cingulate cortex, the left angular gyrus and left inferior frontal gyrus mediating modality dependant differences in agency-related perception processing during the delay detection task. Due to the highly controlled experimental design including active and passive button presses, these results shed new light on sensorimotor and inter-sensory components in the recalibration of action-outcome perception. A novelty of this approach is the ability to compare effects related to the “recalibration process” during adaptation phases and the “recall process” during testing phases. We were able to reveal similarities in brain activation patterns during recalibration and recall (1) in the cerebellum in general and (2) agency-dependant (active > passive) in the right middle/superior frontal gyri, indicating a role of these areas in both processes. Thereby, activations during testing phases due to the recall process were solely dependent on the *adaptation delay* during preceding adaptation phases. As such, our results provide new insights into the different aspects of temporal recalibration.

### Temporal recalibration effect

We found a significant visual, within-modality TRE in active conditions. In this line, temporal recalibration has been observed in a variety of studies^3,4,7–10^. However, a differentiation between sensorimotor and inter-sensory effects was not always included, raising the question of sensorimotor and inter-sensory contributions. This is particularly of interest, since recalibration of temporal perception has been observed in purely inter-sensory contexts^12,13^ and a recent temporal recalibration study found visual TREs to be mainly reliant on the inter-sensory component^7^. In the case of our study, we found no significant interactions in the ANOVA including the movement agency factor, which highlights the main role of the inter-sensory component in temporal recalibration. However, the active and passive TREs were significantly different in directed comparisons for within-modality conditions and only the sensorimotor and inter-sensory components combined were sufficient to induce a significant visual TRE, suggesting a contribution of sensorimotor predictions to visual TREs. This sensorimotor contribution was, however, only found post-hoc, while no interaction effects were found in the ANOVA. Thus, this finding should be interpreted with care and can only indicate a role of sensorimotor predictions in within-modality visual TREs.

We observed significant auditory, cross-modality TREs, with no difference between active and passive conditions. Thus, our data suggest the transfer from vision to audition to be driven by the inter-sensory component. In this line, transfer of inter-sensory recalibration has been shown to occur from audio-visual to audio-tactile and visuo-tactile stimulus pairs^34^. Similar to our results, a recent study found transfer of temporal recalibration from visuo-motor to audio-motor stimulus pairs, mainly reliant on an inter-sensory component^7^. Since no sensorimotor component was evident in the transfer from vision to audition, our results do not support the notion of a supra-modal processing of sensorimotor predictions in temporal recalibration. Similarly, in recent temporal recalibration studies, no sensorimotor transfer of temporal recalibration was observed from audition to vision^7,10^.

### Neural correlates of temporal recalibration

The occurrence of a TRE is a result of updated neural representations of action-outcome relationships during the exposure to unexpected timings of sensory outcomes in adaptation phases. It has been postulated that, before temporal recalibration, the human brain expects sensory outcomes to appear immediately after the action^4^. Thus, the generating and updating process should be especially important for delayed, rather than undelayed outcomes during adaptation phases. Several studies believe the cerebellum to incorporate functions of internal forward models^1,3,24^, including the storing of representations of the external world. In addition, the cerebellum has been shown to be associated with sensorimotor temporal recalibration processes^3,10^. Internal forward models, however, rely on an efference copy of a motor command to predict sensory outcomes^1–3,5^ and can thus only apply for sensorimotor contexts. Yet, the cerebellum has been shown to be associated with temporal processing in purely inter-sensory contexts^35^ and to be involved in the recalibration of sensory spatiotemporal predictions^36^. Moreover, facilitation of cerebellar function has been shown to interfere with sensorimotor and inter-sensory temporal recalibration^11^, raising the question to which extent cerebellum activation can be attributed to a sensorimotor or a more general inter-sensory component. In the case of this study, we found cerebellum activation across active and passive conditions, suggesting that it was driven by the inter-sensory component. The cerebellum activation due to temporal recalibration during adaptation phases might thereby reflect increased processing demands due to storing of newly experienced inter-sensory relationships and the generation of new predictions. Similarly, the cerebellum recruitment during testing phases might reflect the neural demands associated with the retrieval of aforementioned representations. The overlap in activation between adaptation and testing thereby seems to suggest a storage site of neural representations in the lobules IV and V of the cerebellum.

Additionally, we found temporal recalibration to be associated with activation in the hippocampus during testing phases. We propose that during testing phases, the temporal structure of action-outcome pairs is compared to the stored neural temporal representation. This comparator function is decisive on whether the sensory outcome is judged as delayed (mismatch) or undelayed (match). Previous studies found the hippocampus to be involved in sensory evaluation processes, activating due to matches/mismatches in the sensory systems^37–39^. Moreover, the hippocampus has been observed to be sensitive for the temporal structure of stimulation in a temporal match/mismatch task^40^. In addition, in a recent study we found the hippocampus to be involved in temporal recalibration in the auditory domain^10^. Based on these studies and our results, it seems plausible that the hippocampus might incorporate sensory matching functions of a comparator. The increased neural efforts after temporal recalibration might thereby be a result of increased processing demands due to the retrieval of newly learned inter-sensory relationships leading to an increased uncertainty in the comparator process. Since we found the hippocampus activation across both, active and passive conditions, this comparator function seems to operate on an inter-sensory level. The activation cluster in the hippocampus also extends into the amygdala. In previous animal research, it has been shown that temporal prediction errors can trigger memory updating processes in the amygdala in appetitive and aversive association learning contexts^41,42^, suggesting a role of the amygdala in predictive processes. In the human context, studies suggest the amygdala to be involved in the processing of body ownership^43,44^. Similar processes might be affected in our experiment since the delayed sensory outcomes might interfere with the sense of agency regarding the sensory outcomes. Nevertheless, our results indicate an involvement of the amygdala in a non-affective temporal recalibration context.

The neural correlates of inter-sensory temporal recalibration during testing phases discussed above were observed across visual and auditory sensory testing modalities. In addition, there was no interaction between the *adaptation delay* and the *sensory testing modality*. Thus, we found no significant differences in the processing of inter-sensory recalibration between modalities, suggesting largely similar recalibration processing.

### Agency-dependant processing of recalibration

We found differential activation patterns between movement agencies, with increased activation due to active and decreased activation due to passive recalibration. During adaptation phases, this included a single cluster in the prefrontal cortex, extending over the right superior and middle frontal gyri. Increased neural demands due to active recalibration might thereby be a result of the processing of sensorimotor prediction errors occurring due to unexpectedly delayed visual outcomes. Inter-sensory recalibration in passive conditions does not involve sensorimotor predictions, which might explain why this effect is absent during passive adaptation phases. Instead, the reversed effect might rather reflect a perceived temporal synchrony between sensory information from the externally generated button press and the sensory outcome. Interestingly, we found similar regions to be involved in auditory recalibration^10^. However, the lateralisation and direction of effect was reversed, suggesting a modality specific processing.

The same pattern during testing phases revealed activation in the parietal lobe (bilateral postcentral gyri, bilateral superior/inferior parietal gyri), temporal lobe (left middle/inferior temporal gyri) and frontal lobe (right superior/middle frontal gyri, left superior frontal gyrus, bilateral supplementary motor areas, bilateral precentral gyri). The supplementary motor area has been found to be involved in various functions, including action-outcome monitoring^45^. Areas in the right temporoparietal junction were found to be involved in inter-sensory conflict detection^46^ and temporal and parietal areas are proposed to be involved in detecting sensory mismatches^24^. Thus, processing of sensory errors and sensory information evaluation has previously been shown to be associated with a variety of brain areas, including areas we found in this study. In the case of our study, the direction of effect might thereby originate from different neural processes. The increased neural efforts after active temporal recalibration might reflect the increase in task difficulty, originating from a greater uncertainty in newly learned neural action-outcome relationships or competing sensory expectations based on multiple stored internal representations. This effect was absent in passive conditions, indicating activation specific to active button presses. Contrary to that, neural efforts were reduced after passive recalibration. We propose that in testing phases during passive conditions, a delay is detected as a sensory error reflecting asynchrony between tactile and proprioceptive information of the button press and the sensory outcome. After passive recalibration, the sensitivity to delays is reduced; thus, the reduction in neural efforts might reflect less processing of sensory errors. Auditory conditions contributed more to the overall effect strength than visual conditions. This might be a result of more uncertainty during delay detection and novelty effects related to the untrained auditory outcome condition.

Activation in the superior and middle frontal gyri overlapped between adaptation and testing phases, indicating that these areas are not only involved in the processing of recalibration during adaptations, but also during the retrieval during testing. Overall, these agency specific effects might reflect the generation (during adaptation) and use of sensorimotor predictions (during test phases), which are especially relevant when considering sequential behaviour (here the repeated button-presses) under changing conditions (here delayed/undelayed).

### Agency-dependant processing of recalibration in interaction with the outcome modality

The agency-dependent activation pattern further interacted with the sensory testing modality in testing phases. We found the same pattern as described in the previous section during auditory conditions, while the pattern was reversed for visual conditions with decreased activation due to active and increased activation due to passive recalibration. This included activation in the left frontal cortex (left inferior frontal gyrus), left parietal cortex (left angular gyrus) and posterior areas of the cingulate cortex (bilateral posterior/middle cingulate gyri). The left inferior frontal gyrus has been found to be important for multisensory category learning^47^, indicating a processing of multisensory information integration. Similar processing might be involved in our study in case of the transfer from vision to audition, potentially contributing to differential effects in within-modality and cross-modality conditions. The frontal cortex has also been shown to be involved in the processing of sensory predictions and prediction errors^48,49^. This is of interest since we propose sensorimotor recalibration to rely on prediction making processes and prediction errors. A potentially closely related neural function is the processing of sensory errors, repeatedly shown to be associated with the angular gyrus; This includes the awareness of sensory errors^23^ and temporal matching functions^24^. In this line, the angular gyrus was proposed as a supra-modal comparator area^16^. In the case of this study, we found sensorimotor predictions to contribute only to visual TREs in the post-hoc analysis. Thus, decreased neural demands in visual testing phases after active temporal recalibration might reflect a decrease in sensorimotor prediction errors since a prediction error forms the basis of delay detection and less delays were detected after recalibration. Since no sensorimotor contribution was evident in auditory testing phases, the increased neural efforts after recalibration might solely reflect an increase in task difficulty as described in the previous section. We found a significant passive TRE in auditory, but not visual conditions. Thus, while decreased neural efforts in auditory testing phases after recalibration might reflect less processing of inter-sensory errors, as described in the previous section, this seems to be less important in visual testing phases. Instead, the increased neural demands in visual conditions might, although the TRE was non-significant, reflect a slight increase in uncertainty in inter-sensory expectations introduced in adaptation phases. The observed activation pattern also involved activation in the posterior cingulate cortex. The posterior cingulate gyrus is a highly interconnected area within the human brain, proposed as a connector hub^50^. Interestingly, a previous study found comparator functions in the angular gyrus to be functionally connected to areas, including the posterior cingulate cortex^16^; The authors proposed this connectivity to represent the integration of self-referential processing in these areas and the delay detection in the angular gyrus. Similarly, in the case of our study, the cingulate cortex activation might represent a mediation of the complex neural processes in the different testing conditions, rather than specifically incorporating delay detection processes.

### Limitations

A potential limitation of our study lies in the approach of modelling the button presses as whole phases during adaptation. While this is a robust way to model such a fast event train, different processes such as motor planning, movement, delay recognition and delay adaptation contribute to activation of a given condition. As such, the regressors of interest include recalibration-dependent and -independent effects. However, this is no major concern, since we analysed condition specific activations in a comparative manner, and we have no apparent reason to believe general aspects of sensorimotor processing and sensory perception to significantly differ between conditions. Thus, recalibration-independent effects factor out during the statistical analysis. Nevertheless, a potential condition-dependent factor is the attentiveness with which sensory outcomes are perceived. Since active button pressing requires an active involvement, it might help at sustaining attentiveness throughout adaptation phases. Thus, attentiveness might influence TREs more strongly in passive conditions. Although the participants were instructed to perceive every sensory stimulation as watchful as possible and received cues during recalibration, the attentiveness cannot be quantified and can thus not be corrected for. This would, however, affect within-modality and cross-modality conditions and we found a slight difference between active and passive TREs only in within-modality conditions. Thus, it seems unlikely that the sensorimotor contribution we found in active visual TREs is solely explainable by attention.

Another question to consider is how the strength of the recalibration effect is modulated by the length of the adaptation phases or the amount of sensory stimulation in the recalibration process. The experimental procedure in the present study incorporated rather short adaptation phases with frequent switches of experimental conditions. Longer adaptation phases might lead to a more pronounced recalibration effect in both, sensorimotor and inter-sensory recalibration. Moreover, one might assume that the speed at which sensorimotor and inter-sensory interactions are capable of inducing adaptations in the temporal perception of action-outcome relationships is not identical. Thus, the amount of stimulation needed to reach the full potential of recalibration might differ between sensorimotor and inter-sensory adaptation processes.

Finally, a general limitation of yes/no experimental paradigms, including the one used here, is the potential impact of individual decision making on the behavioural results. That is, each response to the delay question is a combination of actual perceptual discriminability and an individual criterion, with which participants judge outcomes to be delayed or not. Issues regarding the impact of individual criteria and response biases on behavioural data have, for instance, been discussed in the context of self-identification tasks in previous research^51^. In case of this study, we cannot distinguish between both effects and thus, reported behavioural recalibration effects might be a combined recalibration of perceptual discriminability and the individual criterion. To disentangle both effects, future studies might want to apply, for example, a two alternative forced choice paradigm, where delayed stimuli are judged against undelayed stimuli, rather than an individual criterion. Similar designs were applied in similar and different contexts in previous studies^52–55^.

## Conclusion

In conclusion, we found active TREs in within-modality, visual conditions to be driven by the inter-sensory component, with a contribution of the sensorimotor component, found post-hoc. While behavioural data suggest that only the inter-sensory component transferred from vision to audition, the fMRI data point to a more complex pattern with several action specific effects, with posterior areas in the cingulate cortex, the left angular gyrus and left inferior frontal gyrus mediating modality dependant differences in agency-related perception during the delay detection task. Our results further suggest the importance of the cerebellum (active and passive) and the middle/superior frontal gyri (active > passive) in the acquisition and recall of new temporal inter-sensory and sensorimotor relationships, respectively. We further found differential effects on matching processes, comparing predicted and perceived sensory outcomes, of active vs. passive movements after recalibration. This was associated with an increase in activation in active and a decrease in activation in passive conditions in parietal (bilateral postcentral gyri, bilateral superior/inferior parietal gyri), temporal (left middle/inferior temporal gyri) and frontal areas (right superior/middle frontal gyri, left superior frontal gyrus, bilateral supplementary motor areas, bilateral precentral gyri). These results provide new insights on different contributions of sensorimotor and inter-sensory predictions in within-modality and cross-modality temporal recalibration and their differential effects on brain activation. Based on these results future research should investigate the relevance of the described effects for more natural sequential behaviour and potentially related dysfunctions in action-outcome monitoring in clinical groups such as schizophrenia^56–58^.

## Data Availability

The data that support the findings of this study are openly available in Zenodo at 10.5281/zenodo.10057006.
